# Risk Factors for Post Laryngectomy Pharyngocutaneous Fistula and Impact of Pharyngeal Suture Type on Fistula Characteristics

**DOI:** 10.22038/ijorl.2024.83054.3798

**Published:** 2025

**Authors:** Javier Gómez-Hervás, Eduardo J Correa, Diego M Conti, Georgia Liva, Esteban Merino-Galvez

**Affiliations:** 1 *Otolaryngology Department, Rafael Mendez University Hospital, Lorca, Spain.*; 2 *Otolaryngology Department, Hospital Comarcal de la Línea de La Concepción, Cádiz, Spain.*; 3 *Doctoral School UAM, Graduate Studies Center, Autonomous University of Madrid. Calle Francisco Tomás y Valiente, No. 2. Cantoblanco University City, 28049 Madrid, Spain.*; 4 *Allergy and Clinical Immunology Research Unit, Department of Microbiology and Immunology, KU Leuven, Leuven, Belgium.*; 5 *Otorhinolaryngology Department, University Hospital of Heraklion, University of Crete, School of Medicine. Crete, Greece.*; 6 *Otolaryngology Department, San Antonio Catholic University of Murcia. Murcia, Spain.*

**Keywords:** Cutaneous Fistula, Laryngectomy, Suture Techniques

## Abstract

**Introduction:**

Pharyngocutaneous fistula (PCF) is the most common complication following total laryngectomy (TL). The factors contributing to its occurrence are still a matter of debate. The impact of suture type has been relatively underexplored. This study aimed to analyze the risk factors associated with PCF and understand how the type of suture influences PCF characteristics.

**Materials and Methods:**

An observational study encompassing all TL procedures was performed between 2005 and 2022 at a secondary care hospital. Sociodemographic and clinical variables widely studied in the literature to identify PCF risk factors were considered. Additionally, the characteristics of fistulas were examined to assess the influence of the suture type.

**Results:**

Seventy TL cases were included. The incidence of PCF was 56.0%. Identified risk factors for PCF included pharyngeal closure type (p=0.001) (RR=13.09), nutritional support type (p=0.001) (RR=13.54), the need for reintervention due to postoperative bleeding (p=0.001) (RR=1.13), and the need for blood transfusion after surgery (p=0.015) (RR=1.20). Regarding the suture type, Modified Connell Suture (MCS) was associated with a later onset of fistula (p=0.014), shorter hospital stay (p=0.001), and early initiation of oral feeding (p=0.009).

**Conclusion:**

PCF occurrence is associated with nasogastric tube use, Lambert closure, postoperative bleeding, and reintervention for bleeding after TL. Moreover, MCS sutures are linked to a shorter hospital stays and early initiation of oral feeding.

## Introduction

Despite the increasing utilization of non-surgical therapeutic approaches for laryngeal tumors, total laryngectomy (TL) remains a fundamental technique for addressing primary laryngeal cancer and recurrent or persistent cases following the failure of other therapies (1). One of the most common complications is the development of a pharyngocutaneous fistula (PCF) after surgery (2). Its presence is correlated with increased morbidity (aspiration-related respiratory infections, malnutrition, and prolonged hospital stay) and delays in the initiation of other oncological therapies (3).

The causes leading to the occurrence of PCF are still debated. Described risk factors include preoperative tracheotomy, low postoperative hemoglobin levels, previous radiotherapy, positive surgical margins, or the presence of positive lymph nodes (2,3). One crucial aspect we consider is the type of suture used for pharyngeal closure, which has been relatively understudied (4). The objective of this study is to identify the risk factors associated with the occurrence of PCF and observe the influence of the type of pharyngeal closure on the occurrence and characteristics of PCF.

## Materials and Methods

*Study Type*

We conducted an observational study in a retrospective cohort, including all patients undergoing total laryngectomy (TL) between 2005 and 2022 in the otolaryngology department of a secondary care hospital. No exclusion criteria were applied. The surgical team remained constant, with surgeons aged between 35 and 62. Antibiotic prophylaxis with amoxicillin-clavulanate was administered from the day of surgery until the seventh postoperative day. When a PEG tube was used, it was inserted by the digestive department on the same day as the TL in the first surgical procedure. Postoperative care followed the same criteria: daily cannula changes and compressive dressing for the first 10 days. If no pharyngocutaneous fistula (PCF) occurred, oral feeding was initiated around the 14th day after surgery. The initial management of PCF was conservative, with antibiotic therapy maintained and feeding sustained via nasogastric or PEG tube. Surgical closure was considered if spontaneous closure did not occur within a month.

*Variables to Assess PCF Risk Factors*

1. Sociodemographic variables included age, sex, and year of intervention.

2. Patient clinical variables included smoking, alcohol consumption, gastroesophageal reflux, diabetes, and dyslipidemia.

3. Tumor-related variables included size, location, lymph node involvement, distant metastasis, need for tracheotomy, radiotherapy, and prior partial surgery before TL.

4. Surgical intervention-related variables included cervical clearance, type of pharyngeal closure, drainage type, postoperative bleeding, transfusion after TL, affected surgical margins, and nutritional type.

*Variables for Comparing Pharyngeal Closure Types*

Suture Types: There are two major types—interrupted and continuous. Interrupted sutures, like Lambert, are performed submucosally, aiming to evert wound edges with submucosal penetration and T closure (5). Continuous sutures include Modified Connell Suture (MCS), a continuous submucosal suture with a double throw, starting from each end of the T and reaching the midline, then returning to the initial point (VIDEO1) (4). Additionally, surgical time, months of survival, day of PCF onset and closure, total days with fistula, day of oral feeding initiation, and hospital stay were calculated for both pharyngeal closure types.

*Statistical Analysis*

 Data were analyzed using SPSS v23. The normal distribution of the sample was verified (Shapiro-Wilks). The Chi-square test was used for qualitative variables. Also, Levene's test and Student's t-test were used for quantitative variables. A p-value < 0.05 was considered statistically significant.

*Ethics Committee*

The study received approval from the hospital’s Research and Ethics Committee. Registration ID: CEI-2023-02-09.

## Results

A total of 70 total laryngectomies were performed between 2005 and 2022. Only 5.3% were women. The mean age was 63.3 years (±9.40). The incidence of pharyngocutaneous fistula (PCF) was 56%. Surgical closure was required in 14%. Table 1 illustrates the relationship between the occurrence of PCF and the studied variables. Based on the table, we can consider the following as risk factors: pharyngeal closure type (p=0.001), nutritional type (p=0.001), the need for reintervention due to postoperative bleeding (p=0.001), and the need for blood transfusion after surgery (p=0.015). No significant differences were found in the mean age of patients with PCF, 63.22 (±9.59), and without PCF, 62.54 (±8.44), p= 0.761.

**Table 1:** Relationship between Study Variables and the Occurrence of FCF

**Study Variable**	**PCF**					**No PCF**				**p-value**	**RR (CI 95%)**
	**N**			**%**		**N**		**%**			
Sex											
Male	41			61,2		26		38,8		0,298	
Female	1			25		3		75			
Smoking											
Yes	39			61.9		24		38,1		0.118	
No	7			71,4		2		28,6			
Alcohol Consumption											
Yes	28			66,7		14		33,3		0.137	
No	13			46.4		15		53.6			
GERD											
Yes	1			33,3		2		66,6		0.563	
No	41			60.3		27		39,7			
Dyslipidemia											
Yes	6			42,9		8		57,1		0.230	
No	35			62.5		21		37,5			
Diabetes											
Yes	4			40		6		60		0.298	
No	38			62.3		23		37,7			
Metastasis											
M0	40			59,7		27		40.3		0.783	
M1	1			50		1		50			
Prior Tracheostomy											
Yes	22			56.4		17		43,6		0.636	
No	20			62.5		12		37.5			
PriorLaryngealSurgery											
Yes	7			46.7		8		53.3		0.382	
No	33			61.1		21		38.9			
Prior Radiotherapy											
Yes	9			60		6		40		0.940	
No	33			58.9		23		41.1			
Pharyngeal Closure											
Lambert	30			87.7		5		14.3		0.001	13,09
MCS	11			31.4		24		68.6			(4,00-42,84)
Neck Dissection											
Yes	40			58.8		28		41.2		0.787	
No	2			33.3		1		66.7			
Drainage Type											
Aspirative	28			66.7		14		33.7		0.314	
Penrose	13			54.2		11		45.8			
Post Bleeding											
Yes	10			100		0		0		0.001	1,13
No	30			50.8		29		49.2			(1,06-1,18)
Transfusion after TL											
Yes	15			83.3		3		16.7		0.015	1.20
No	26			50		26		50			(1,05-1,77)
Surgical Margins											
Affected	9			64.3		5		35.7		0.604	
Free	30			56.6		23		43.4			
Nutricional Type											
PEG Tube	16			38.1		26		61.9		0.001	13.54
Nasogastric Tube	25			89.3		3		10.7			(3,51-52,23)
Post Hemoglobin											
Menor de 12 g/dl	22		55.0			13		52.0		0.813	
Mayor de 12 g/dl	18		45.0			12		48.0			
						
	PCF					No PCF				Valor de p	
	N	%			AR	N	%		AR		
Tumor size											
T1	0	0				0	0			0.06	
T2	0	0			-2.17	3	10.7		2.17		
T3	19	45.2			-0.39	14	50		0.39		
T4	23	54.8			-1.27	11	39.3		1.27		
Nodal Involvement											
N0	25	59.5			-1.12	21	72.4		1.12	0.536	
N1	5	11.9			1.26	1	3.4		-1.26		
N2	10	23.8			0.31	6	20.7		-0.31		
N3	2	4.8			0.27	1	3.4		-0.27		
Tumor Location											
Glótico	8	19,0			-0.52	7	24.1		0.52	0.471	
Hipofaringe	6	14.4			1.51	1	3.4		-1.51		
Subglótico	4	9.5			0.98	1	3.4		-0.98		
Transglótico	13	31.0			-0.61	11	37.9		0.61		
Supraglótico	11	26.2			-0.45	9	31.0		0.45		


Regarding the degree of association between variables, we observed that MCS closure and PEG tube presented a strong association with the absence of PCF, RR 13.09 (4.00-42.84) and RR 13.54 (3.51-52.23), respectively. On the other hand, the need for reintervention due to bleeding and the need for blood transfusion after surgery showed a weak association, RR 1.13 (1.06-1.18) and RR 1.20 (1.05-1.77), respectively.

Table 2 describes the clinical characteristics of patients according to the type of suture used. It was found that MCS closure resulted in a later onset of fistula (p=0.014), a shorter hospital stay (p=0.001), and an early initiation of oral feeding (p=0.009).

**Table 2:** Relationship between Pharyngeal Closure and PCF Characteristics

	**Pharyngeal Closure Type**	**N**	**Mean**	**Standard Deviation**	**p-value**
Surgical Time (hours)	Lambert closure	12	6,21	1,90	0,069
	MCS	9	5	0,71	
Postoperative Day of PCF Onset	Lambert closure	29	7,17	4,26	0,014
	MCS	10	11,20	4,21	
Day of Spontaneous Closure	Lambert closure	27	53.15	47,57	0,338
	MCS	6	33,50	25,25	
Duration of the PCF (days)	Lambert closure	29	57,07	76,18	0,432
	MCS	10	36,10	56,96	
Day of Oral Feeding Initiation	Lambert closure	31	49,84	46,28	0,009
	MCS	34	23,41	31,37	
Hospital Stay (days)	Lambert closure	34	38	19,84	0,001
	MCS	35	21,80	9,13	
Survival (months)	Lambert closure	30	103.83	112,11	0,199
	MCS	26	71.46	63.49	

PCF: Pharyngocutaneous Fistula. MCS: Modified Connell Suture

## Discussion

Pharyngocutaneous fistula (PCF) poses a significant challenge following total laryngectomy (TL), increasing morbidity and delaying adjuvant oncological therapies (6). The fistula arises from the dehiscence of the pharyngeal closure suture, and the type of technique used during closure and the presence of foreign material (nasogastric tube) in the surgical bed can be decisive factors in the occurrence of this complication (4,7). In our sample, MCS suture and the systematic use of a PEG tube showed a strong association with the non-appearance of PCF, with RR=13.09 (4.00-42.84) and RR=13.54 (3.51-52.23), respectively. The incidence of PCF after TL varies widely in the literature (3-65%) (2,3,5,8). Busoni et al^9^. note an increased incidence in recent years due to the growing application of TL as a salvage technique. This variability may be attributed, among other factors, to the lack of a uniform criterion for defining the fistula. While some authors, like us, consider even small dehiscences as fistulas, others require the profuse discharge of material for classification. Thus, our PCF incidence is at the higher end of the spectrum (56%), yet our surgical closure rate (14%) is lower than in other series (24-44%) (2,10).

^.^

*Risk Factors for PCF*

Risk factors for PCF are a topic of ongoing debate in the scientific literature and need a consensus (2). 

*Systemic Diseases*

According to some researchers, systemic diseases may impact neopharyngeal healing. Boscolo-Rizoo et al. (10) found statistical significance associated with diabetes and hypoalbuminemia, whereas Wang et al. (2) identified significance in COPD and heart diseases, though not in diabetes. Interestingly, none of the systemic diseases we studied showed significant results. These outcomes were echoed by other authors as well (11-13).

*Tumor Size*

In their meta-analysis, Wang et al. (2) found a correlation between tumor size and the onset of PCF, likely due to the larger resection area and reduced residual mucosa. In our study, we approached statistical significance (p=0.06), which could be strengthened with a larger sample size. Our adjusted residuals also support the observations made by Wang et al (2).

*Nutritional Support Type*

We know that dietary choices play a crucial role in predicting complications post-TL (7). Traditionally, NG tubes have been the go-to after TL, reserving PEG tubes for long-term PCF management (14). However, our findings hint at the possibility of considering a proactive approach with PEG tubes, even on the same day as TL. Early studies advised caution due to limited study quality (14,15). Jack et al. (14) suggest prophylactic PEG use for advanced-stage tumors or those with prior radiotherapy. Later, the UK's head and neck cancer nutritional management guideline proposed considering PEG tube feeding when oral intake is expected to be impossible for over 4 weeks (16). They also introduced a paradigm shift, advocating for prophylactic PEG tube placement linked to optimal nutrition and fewer complications (17).Recent research supports this, highlighting prophylactic PEG tube use as a safe, swift, cost-effective, and well-tolerated method that reduces PCF incidents (18,19). Cristian et al. (18) noted a drop from 26% to 8% in PCF occurrence with preventive PEG tube use.

*Surgery and Postoperative Blood Transfusion*

In our sample, reintervention for postoperative bleeding and the need for blood transfusion were weakly correlated with PCF. It may be attributed to tissue damage upon interrupting the healing process, facilitating infection upon reopening the wound (20). We have yet to come across other studies that have previously explored these risk factors. However, in line with our findings, we have encountered studies linking intraoperative bleeding (21), intraoperative blood transfusion (22), and postoperative hemoglobin levels to the onset of PCF (23).

*Pharyngeal Closure Type and PCF Characteristics*

Only some studies delve into this aspect. This scarcity may stem from limited available data, as the operative report must often include the pharyngeal closure type. Consequently, there needs to be a consensus on which closure type to use for the neopharynx post-laryngectomy, largely due to its under-researched nature and the heterogeneous results across various studies(24).

*Vertical vs. Horizontal Closures*

Vertical closures, divided into straight and T closures, are the most commonly used. Horizontal closures are typically reserved for small pharyngeal defects. In a meta-analysis, Chotipanich et al. (24) observed that horizontal closures result in lower tension and lower rates of PCF. However, the authors only included two studies in their analysis, suggesting that the results may be limited. In our practice, we consistently perform T-shaped vertical sutures, which, in our opinion and that of other authors (25), ensure safe tumor resection, particularly in advanced T-stage cases. Additionally, both T-shaped and horizontal sutures are associated with better swallowing outcomes and a lower rate of complications such as diverticula (25).

*MCS and Lambert Suture*

In our experience, using MC sutures was linked to a reduced incidence of PCF. Existing literature generally supports the idea that continuous sutures, like MC, lead to lower rates of PCF by distributing tension more evenly (24). This notion is echoed in studies like that of Avci et al (26), which, similar to ours, found MC sutures to be effective in lowering the risk of PCF. Similarly, Lemaire et al. (27) developed a predictive scale for PCF, where discontinuous sutures were identified as a risk factor. Furthermore, our study revealed that patients receiving MC sutures had shorter hospital stays and quicker initiation of oral feeding. This results from the lower PCF incidence among patients in this group. However, we have yet to find comparable studies to support these additional findings.

*Limitations*

This observational study, while revealing strong associations, does not imply causation. Randomized controlled trials are lacking in this field and are deemed necessary. Technical differences between surgeons were not considered, and future studies should explore the impact of the surgeon's expertise.

## Conclusion

MCS pharyngeal closure and using a PEG tube were strongly associated with the absence of PCF. Reintervention for postoperative bleeding and the need for blood transfusion after TL weakly correlated with PCF occurrence. Compared to Lambert closure, MCS closure was associated with earlier PCF closure, shorter hospital stays, and earlier initiation of oral feeding.
